# Mastery‐Oriented or Outcome‐Oriented Help? How Recipient Ethnicity and Task Difficulty Shape Children's Helping Behavior

**DOI:** 10.1111/desc.70071

**Published:** 2025-09-08

**Authors:** Jellie Sierksma, Astrid M. G. Poorthuis

**Affiliations:** ^1^ Department of Psychology Utrecht University Utrecht the Netherlands

**Keywords:** ethnic attitudes, ethnicity, helping behavior, learning, stereotypes, task difficulty

## Abstract

Teachers and parents often scaffold children to help others. Not all help is equally beneficial, however. We know very little about the ways in which children distribute different types of help. Across three preregistered studies, we examined when children provide others with help that can hamper learning (outcome‐oriented help, e.g., correct answers) and when they provide beneficial help (mastery‐oriented help, e.g., hints). Dutch children (total *N* = 532, 7–12 years) helped peers from different ethnic groups with difficult and easy tasks. In all three studies, children provided less mastery‐oriented help when tasks were difficult. Children also gave less mastery‐oriented help to Black peers when tasks were difficult, but only when they liked this ethnic group (Studies 1 and 2). Conversely, children helped White and Middle‐Eastern children similarly (Study 3). Children might thus not always provide help that is beneficial to recipients in the long run, particularly when things get difficult and recipients belong to other ethnic groups they like.

**Summary:**

- We examined when children (7–12 years) give peers outcome‐oriented help (e.g., correct answers) and when they provide mastery‐oriented help (i.e., hints).
- Across the three preregistered studies, children provided less mastery‐oriented help when tasks were difficult compared to easy.
- For difficult tasks, children gave less mastery‐oriented help to Black peers when they liked this ethnic group, but helped White and Middle‐Eastern children similarly.
- Children thus provide less beneficial help when things get difficult and recipients belong to ethnic groups they like.

## 1 Introduction

Children often help each other. They help informally, for example, at the playground or at home, and in more formal settings, for example, when peer‐to‐peer helping is used as a learning strategy at schools (Tenenbaum et al. 2020). Providing and seeking help is generally considered to have many benefits: It makes children happy, popular in the classroom, and do better at school (Aknin et al. 2012; Ryan et al. 2001; Warden and MacKinnon 2003), and it is often assumed to foster social cohesion (Killen and Verkuyten 2017; Over 2018; Sierksma 2023b; Taylor 2020). Yet, not all help is equally beneficial. Some help, for example, does not provide recipients with the opportunity to develop their skills (e.g., taking over and providing correct answers), while other types of help do (e.g., providing strategies and asking questions; Leonard 2021; Nadler 2002; Pomerantz et al. 2007; Sierksma 2023a). Developmental research to date focused on *whether* or *how much* children help, but overlooked *how* children help peers. Across three preregistered studies, we therefore examined how children (7–12 years) distribute different types of help to peers and whether task difficulty and the recipient's ethnicity impact their helping.

Understanding how children help is important because providing less beneficial types of help has negative downstream consequences for learning and motivation (Park et al. 2023; Pomerantz et al. 2007; Sierksma 2023a; Sierksma and Brummelman 2025; Sierksma and Shutts 2025). If, for example, children provide less beneficial help to peers who belong to marginalized groups (e.g., low SES, children with immigrant backgrounds), this could exacerbate existing gaps in achievement and wellbeing (Brummelman and Sedikides 2023; Sierksma 2023a; Umaña‐Taylor 2016). Moreover, caretakers and schools often encourage prosocial behavior in children because they believe this has benefits for learning and development (Dahl 2015; Kilian et al. 2007). By understanding when children provide help that benefits learning and when they do not, we can foster positive outcomes of exchanges of help during childhood.

### 1.1 Different Types of Help

Children are motivated to help others from a young age onward: By 14 months of age, they hand over out‐of‐reach objects (Warneken and Tomasello 2006) and provide help to peers who want to play a game (Hepach et al. 2017). Although children, like adults, provide help for many different reasons, one prominent motivation seems to be that they feel for others and want to alleviate their needs (Decety et al. 2016; Eisenberg et al. 2006; Martin and Olson 2015; Paulus 2014; Warneken and Tomasello 2006). Children as young as two, for example, experience positive emotions when someone's need is alleviated, also when others provide that help (Hepach et al. 2012; Hepach and Tomasello 2020) and feelings of empathy and sympathy foster helping during early and middle childhood (Eisenberg et al. 2006; Sierksma et al. 2015; Vaish et al. 2009). Children as young as five also help paternalistically: Giving others help they need to complete a goal, even if they ask for other types of help (i.e., when people ask for a broken cup, they give one that is not broken; Martin et al. 2016). When children help, they are thus motivated to solve other people's needs.

Different types of help solve different kinds of needs. Consider, for example, a classroom in which children are working on math problems and two classmates are paired to help each other. How will they help? Perhaps the classmate decides to provide correct answers. Such *outcome‐oriented* help alleviates needs in the short run, but often does not promote the development of skills in the long term. The classmate might also decide to provide strategy hints. Such *mastery‐oriented* help does not provide immediate relief of a need, but has long‐term benefits because it fosters the development of skills, and recipients are more likely to solve similar problems without help in the future (Nadler and Chernyak‐Hai 2014; Pomerantz et al. 2007; Sierksma 2023a). Indeed, research shows mastery‐oriented help produces higher achievement at school than outcome‐oriented help (Ryan and Shim 2012; Schenke et al. 2015).

Adults often differentiate in the type of help they provide (Bonawitz et al. 2011; Grolnick 2002; Klahr and Nigam 2004; Nadler 2002; Nadler and Chernyak‐Hai 2014; Pomerantz et al. 2007), and children, like adults, seem to understand that not all help is equal. In one study (Raport et al. 2024), for example, 2‐ and 3‐year‐old children got to pick a helper for a puppet who wanted to learn how to do something (e.g., pour water into a cup). Children were more likely to pick a helper who showed the puppet how to do it compared to a helper who simply completed the task for the puppet. Similarly, in interviews, school‐aged children say that they think that mastery‐oriented help is more effective than outcome‐oriented help (Barnett et al. 1982), and by age 7 children think that when peers receive mastery‐oriented help, they are smarter and will learn more compared to peers who receive outcome‐oriented help (Sierksma 2023a).

When children are on the *receiving* end, they also respond differently to outcome‐oriented and mastery‐oriented help (Shell and Eisenberg 1996). Children, for example, dislike receiving outcome‐oriented help more (Sierksma and Brummelman 2025), and it can lower their motivation to persist (Leonard 2021). And when children seek help with learning, they also prefer mastery‐oriented help rather than outcome‐oriented help (Newman and Schwager 1995). Children thus understand mastery‐oriented help is most beneficial for learning and prefer such help themselves. However, will they also provide the most beneficial help to others?

### 1.2 How Do Children Distribute Different Types of Help?

When children have to decide how to help others, they might foremost consider which type of help alleviates a recipient's need best. Mastery‐oriented help calls on a recipient's ability to use that help successfully. That is, if recipients are not able to learn from the hints they got or the questions someone asked, such help does not alleviate their need and might even aggravate it. In these instances, outcome‐oriented help could be seen as a better strategy to alleviate need. The type of help children provide thus likely depends on whether they expect others to be able to profit from mastery‐oriented help. Here, we focus on two factors that, previous research suggests, impact children's expectations about other people's ability to master new skills and knowledge: Task difficulty and whether recipients belong to marginalized groups in their society.

First, whether children expect others to profit from mastery‐oriented help may depend on how difficult tasks are. When a peer works on a task that is hard, children might not be certain that mastery‐oriented help benefits them (e.g., “will they be able to figure it out when I give them a hint?”) and will thus be less inclined to help that way. Although children might then provide outcome‐oriented help with good intentions, it takes away the opportunity to learn for recipients precisely at tasks for which they need to develop skills. Giving more outcome‐oriented help on difficult tasks can then lead to negative consequences: It can lower children's achievement at school (Ryan and Shim 2012; Schenke et al. 2015) and impede the learning goals teachers might have in mind when they implement practices such as peer‐to‐peer‐helping (Tenenbaum et al. 2020). Previous research suggests that children adjust the information they provide to others based on task complexity and the recipients’ competence (Baer and Friedman 2018; Bridgers et al. 2020; Gelman et al. 2013; Ronfard and Harris 2018) and that children provide *more* help on difficult tasks (Bennett‐Pierre et al. 2018). One study also showed that children give less mastery‐oriented help when they know the task is harder for a recipient because they are less competent (Sierksma 2023a). We extend this research and examine whether task difficulty also guides the types of help children give. We hypothesize that children will provide less mastery‐oriented help when tasks are difficult compared to easy.

Second, *whom* children are helping may shape whether they think others benefit from mastery‐oriented help. People from disadvantaged backgrounds (i.e., low‐SES, migration background) are often perceived as vulnerable (Bauer et al. 2021; Brannon 2023; Silverman et al. 2023) and lower in intellectual ability (Copping et al. 2013; Durante et al. 2017; Rowley et al. 2007; Sierksma et al. 2018). When children help others who belong to marginalized groups, societal stereotypes may amplify their uncertainty about whether mastery‐oriented help alleviates need. Children might, for example, assume that peers who belong to marginalized groups are less intelligent or pity them. As a consequence, children could provide more outcome‐oriented help compared to mastery‐oriented help to solve their needs.

There is social psychological research that suggests societal stereotypes can lead adults to help in paternalistic ways (e.g., proving more help because they pity certain groups; Cuddy et al. 2007; Fiske et al. 2002). And adults provide less mastery‐oriented help to recipients who belong to lower status outgroups (Nadler and Chernyak‐Hai 2014; see also Van Leeuwen and Zagefka 2017). Previous research shows recipient's ethnic or racial group can also impact how much children want to help others (Katz et al. 1976; Weller and Hansen Lagattuta 2013). In one study, Dutch children (10–13) helped Black peers *more* when they endorsed the societal stereotype that this group was less intelligent (Sierksma et al. 2018), presumably because this stereotype led them to assume Black peers needed more help. Based on these findings and social psychological theory (Fiske et al. 2002; Nadler 2002), we hypothesize that children might provide less mastery‐oriented help to peers who belong to marginalized groups. This bias likely only emerges, however, when tasks are difficult and there is uncertainty about whether mastery‐oriented help is beneficial.

### 1.3 The Present Research

We examined the impact of task difficulty and recipient's ethnicity on children's helping behavior in three experimental studies. In all studies, children decided what type of help (i.e., outcome‐oriented or mastery‐oriented help) to provide to peers who were working on difficult and easy word puzzles. We preregistered two hypotheses. First, we expect that children will provide less mastery‐oriented help when tasks are difficult compared to easy. Second, we expect that children provide less mastery‐oriented help to peers belonging to marginalized ethnic groups (vs. nonmarginalized groups) when tasks are hard, but not when tasks are easy.

We tested a wide age range (7–12‐year‐old children) to be able to detect developmental differences in children's helping. We did not test younger children because studies suggest that before the age of 7 children have trouble understanding that not all help leads to positive outcomes (Qiu et al. 2024; Sierksma 2023a). We chose to examine two specific types of help (i.e., hints and correct answers), as these are frequently studied in previous research (Newman and Schwager 1995; Shell and Eisenberg 1996; Sierksma 2023a) and common in school contexts.

We also explored the impact of children's ethnic attitudes and ethnic stereotypes about intelligence, as sometimes recipient ethnicity only impacts prosocial behavior when children endorse negative views about outgroups (O'Driscoll et al. 2021; Sierksma et al. 2018), but other times attitudes do not play a role (Renno and Shutts 2015). Another exploratory measure concerned the type of help children themselves wanted to receive on difficult and easy tasks. Understanding whether children's own preferences impact how they help others could provide insight into why they differentiate in their helping.

All three studies were conducted in the Netherlands between 2022 and 2024. We included three ethnic groups: White Dutch (Studies 1–3), Black (Studies 1 and 2), and Middle‐Eastern (Study 3). People with Black and Middle‐Eastern backgrounds represent the largest immigrant groups in the Netherlands with a non‐Western background. People with a Middle‐Eastern background most often migrated from Turkey and Morocco for work (starting around 1950). Black people often migrated from Surinam and the Dutch‐Caribbean, which are former colonies of the Netherlands. People from Surinam and the Dutch‐Caribbean are often more proficient in the Dutch language than people from Turkey and Morocco (Dagevos et al. 2022). People from Turkey and Morocco most often identify as Muslim, whereas people from Surinam and the Dutch‐Caribbean identify with various religions (Dagevos et al. 2022; Huijnk 2018). Both groups report high levels of discrimination (Dagevos et al. 2022), and research shows that Dutch adults and children are often more positive about Black people than people from Turkey and Morocco (but most positive about the in‐group; de Bruijn et al. 2020; Verkuyten and Kinket 2000).

## 2 Study 1

### 2.1 Method

#### 2.1.1 Participants

We determined our sample size a priori using summary‐statistics‐based power analysis (Murayama et al. 2022; see preregistration) and preregistered a sample size of 136 children. However, data collection took place in a science museum for a fixed time, and we included all children who wanted to participate (but excluded those who did not finish (*n* = 6) and one child outside our age range). The science museum is located in Amsterdam, but visited by people from all over the Netherlands. The final sample consisted of 169 children between 7 and 12 years (*M* = 10.45, SD = 1.22, eight parents did not report age; 50.9% boys, 49.1% girls) of which most children were 10 years (*n* = 58), 11 years (*n* = 50), or 12 years (*n* = 32). Parents reported children's ethnicity and indicated that 74% of these children were native Dutch, 6.5% indicated another ethnicity and 19.5% did not report ethnicity. The median annual gross household income fell in the category €59.000–€70.500 (16.3%), and ranged from €11.500–€94.000 or more (40.2% did not disclose this information; median household income was €36.000 in 2023 in the Netherlands, CBS 2024). The modal educational level of parents was higher professional education (Parent 1: 46.2%, Parent 2: 36.4%; 24.9% did not report this information). As preregistered, all children were included in the final sample (the pattern of results below is the same when only native Dutch children are included). Signed parental consent was obtained for all children, and children provided verbal assent.

The data and code necessary to reproduce the analyses presented here are publicly accessible: https://osf.io/pmzct/. Study designs, hypotheses, and data‐analysis plans were preregistered (see: https://osf.io/mv8nf).

#### 2.1.2 Design

A 2 (difficult vs. easy puzzles) by 2 (White vs. Black recipients) within‐subjects design was used. Each child helped a total of 12 children, and the order of the conditions was randomized across participants. When children were done helping, they answered two questions about their own help preferences. Then, ethnic attitudes and stereotypes about intelligence were measured (the order was counterbalanced). Recipients of help were gender matched, and children were told they were in the same grade as them.

#### 2.1.3 Procedure and Measures

Children were tested in a separate room in the museum. They worked independently on a laptop and received instructions via prerecorded audio.

First, we told participants they were going to help some children with word puzzles. We focused on a language‐based task because these are common at school, and stereotypes can impact children's inferences in the language domain (Kinzler and DeJesus 2013). Children saw pictures of 12 children (gender matched, 6 Black and 6 White) taken from The Child Affective Facial Expression set (LoBue and Thrasher 2014) and were told these children were in the same grade as they were. Participants then heard: “The children will work on word puzzles. They will do 12 in total, and the puzzles are somewhat similar. But some word puzzles are easy and others are difficult.” We informed participants that the children could receive help for the first puzzles, that the computer would decide when children could receive help and that they could only receive help once.

##### 2.1.3.1 Practice Phase

When children had to provide help, they would hear a bell ringing. We told children this was their sign to provide help and they were free to decide what type of help to provide: They could click on the orange button to provide a hint (“then children receive an explanation about the word puzzle and can learn how to solve the word puzzles”) or click on the green button to provide the correct answer (“then the child can continue to the next puzzle”). We provided an explicit description of each type of help and highlighted positive consequences so children did not feel pressured to pick one type of help (Sierksma 2023a). Then participants practiced which button to click when they wanted to provide hints and answers (and received performance feedback if they made a mistake).

##### 2.1.3.2 Puzzle Difficulty

**FIGURE 1:**
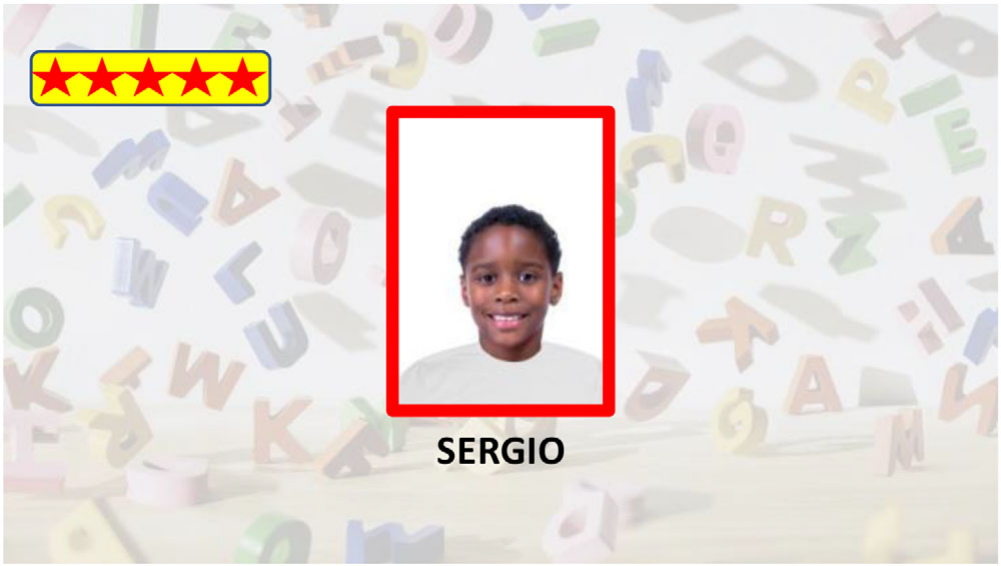
Example of what participants saw when they could help a Black peer with a difficult puzzle.

We emphasized that the word puzzles were all similar to make sure children understood that learning how to solve one of the puzzles was also helpful for solving other word puzzles. We told children that puzzles did vary in difficulty. For difficult puzzles we said: “If you're allowed to help someone with a difficult word puzzle, you'll see this picture (i.e., image with five red stars). All five stars will be red.” And for easy puzzles we said: “If you're allowed to help someone with an easy word puzzle, you'll see this picture (i.e., image with one red star and four white stars). Only one star will be red” (see Figure 1).

##### 2.1.3.3 Helping

Participants were instructed to listen carefully, and when they heard the bell, they could provide help. And that they would help a total of 12 children. Participants then saw 12 different children who were working on easy or difficult word puzzles. When the bell rang, the narrator said: “This is [name child]. He/She is working on a difficult/easy word puzzle. You can now decide what type of help you want to provide”. Typical Dutch and Surinamese first names were used. To analyze helping behavior, we created a sum score for each within‐subjects cell (hints were coded as 1, correct answers were coded as 0; scores ranged from 0 to 3, higher scores indicate more hints).

After helping all children, we told participants we had some remaining questions for them.

##### 2.1.3.4 Help Preferences

We asked participants, “When you are working on a [difficult or easy] puzzle, would you rather receive a hint or the correct answer?”. Participants answered these two questions by clicking on the orange “answer” button or the green “hint” button.

##### 2.1.3.5 Ethnic Attitudes and Intelligence Stereotypes

We used The Child Affective Facial Expression set (LoBue and Thrasher 2014) for both measures. Each participant saw 6 pairs of children of which one was White (coded “1”) and the other was Black (coded “0”) and asked to pick which target they liked more (forced choice). To reduce social desirability, we included two filler trials (one with two Black children and one with two White children; these were not analyzed). Due to a programming mistake, 57 girls rated a total of 36 pairs, whereas all boys rated the preplanned 6 pairs. We included all responses by creating an average (scores above 0.5 indicate children liked White targets more, scores below 0.5 indicate they liked Black targets more). The same procedure was repeated to measure intelligence stereotypes, but participants were asked to indicate which target child they thought was smarter.

Then children were thanked, debriefed, and received a certificate for their participation.

#### 2.1.4 Analyses

Linear mixed models were used in R^1^ (nlme package; Pinheiro and Bates 2024) to examine the data including a random intercept, contrasts for task difficulty (easy “1” vs. hard “−1”; random slope) and recipient's ethnicity (White “1” vs. Black “−1”; random slope), and an interaction between task difficulty and recipient's ethnicity. Exploratory analyses for children's age, ethnic attitudes, and stereotypes about intelligence were conducted by including a standardized continuous score (main and interaction effects). For gender we included a contrast (“1” for boys, “−1” for girls). Note that we also tested children's helping against the midpoint for each cell for each study and report results in the Supporting Information.

### 2.2 Results

#### 2.2.1 Preregistered Analyses

##### 2.2.1.1 Ethnicity and Difficulty

There was a main effect of task difficulty: Children provided less mastery‐oriented help when puzzles were difficult as opposed to easy (*p* < 0.001; easy puzzles *M* = 2.63, SD = 0.80; difficult puzzles *M* = 1.25, SD = 1.23). Ethnicity of the recipient of help did not influence the type of help children gave (*p* = 0.429), and there was no interaction of ethnicity and puzzle difficulty (*p* = 0.155). Children's age (all *p*s > 0.053) and gender all *p*s > 0.240) did not influence their helping (see Supporting Information for details). See Table 1 for an overview of the results.

**TABLE 1:** Overview of results for all three studies.

	Study 1		Study 2		Study 3	
	*β* (SE)	95% CI	*β* (SE)	95% CI	*β* (SE)	95% CI
Difficulty	0.55^***^ (0.05)	0.46, 0.65	0.59^***^ (0.04)	0.50, 0.68	0.54^***^ (0.05)	0.45, 0.63
Ethnicity	0.01 (0.02)	−0.02, 0.04	0.03 (0.02)	−0.01, 0.06	−0.02 (0.02)	−0.06, 0.03
Difficulty X Ethnicity	−0.02 (0.02)	−0.05, 0.01	−0.02 (0.2)	−0.06, 0.01	−0.01 (0.02)	−0.05, 0.03

*** *p* < 0.001.

#### 2.2.2 Exploratory Analyses

##### 2.2.2.1 Ethnic Attitudes and Intelligence Stereotype

Children liked Black targets more than White targets (*M* = 0.44, SD = 0.22, one sample *t* test against neutral midpoint of the scale, *t*(167) = −9.48, *p *< 0.001, *d* = −0.27, CI 95% [−0.42, −0.11]). Children were more likely to say Black children were smarter compared to White children (*M* = 0.40, SD = 0.23, one sample *t* test against neutral midpoint of the scale, *t*(166) = −5.58, *p *< 0.001, *d* = −0.43, CI 95% [−0.59, −0.27).

There was a main effect of ethnic attitudes (*β *= −0.09, SE = 0.03, *p *= 0.013, 95% CI [−0.16, −0.02]) and a significant interaction for recipient's ethnicity and children's ethnic attitudes (*β *= −0.03, SE = 0.02, *p *= 0.046, 95% CI [−0.06, −0.00]). Simple slope analyses showed that when children scored relatively high on the ethnic attitudes measure (1 SD above the mean; i.e., liking White targets more), they helped White and Black recipients similarly, *β* = −0.02, SE = 0.02, *p* = 0.393, 95% CI [−0.06, 0.02]). When children scored relatively low on ethnic attitudes (1 SD below the mean; i.e., liking Black targets more) they provided less mastery‐oriented help to Black compared to White recipients, *β *= −0.04, SE = 0.02, *p *= 0.049, 95% CI [0.00, 0.08]. Thus, only when children were very positive about Black peers did they provided less mastery‐oriented help to them compared to White peers.

Stereotypes about intelligence did not influence children's helping behavior (all *p*s > 0.150; see Supporting Information for details).

##### 2.2.2.2 Help Preferences

For easy puzzles, a majority of children preferred to receive mastery‐oriented help (90.5%) rather than outcome‐oriented help (binomial test, *p* < 0.001). For difficult puzzles, there was no clear tendency to prefer either type of help (mastery‐oriented help: 52.7%, binomial test *p* = 0.538). Children's age was not correlated with their help preferences (easy puzzles: *r* = 0.14, *p* = 0.088, difficult puzzles, *r* = −0.03, *p* = 0.742). Children's preferences influenced the type of help they gave: Children provided more mastery‐oriented help when they preferred such help themselves on easy puzzles (*β* = 0.61, SE = 0.06, *p* < 0.001, 95% CI [0.48, 0.74]) and difficult puzzles (*β* = 0.47, SE = 0.06, *p* < 0.001, 95% CI [0.34, 0.59]). Children's help preferences did not influence how they helped recipients of different ethnicities (i.e., there were no interactions, all *p*s > 0.178).

### 2.3 Discussion

Study 1 showed that task difficulty impacts the type of help children provide. As hypothesized, children provided less mastery‐oriented help when tasks were difficult compared to when tasks were easy. We also found partial support for our second hypothesis: Children were less inclined to provide mastery‐oriented to Black peers (vs. White peers), but only when they evaluated Black peers very positively. Exploratory analyses further showed that children helped in line with the type of help they liked to receive themselves. Children thus seem less likely to provide beneficial help when things get difficult or when recipients belong to ethnic groups they like. The findings for recipient's ethnicity, however, deviated from our hypothesis (i.e., they only emerged for children who really liked Black peers and were not specific to difficult tasks), and the analyses for ethnic attitudes were exploratory, and the effects were small. We therefore conducted a second study to assess the robustness of these findings.

## 3 Study 2

The design of Study 2 was similar to Study 1, but we also made some improvements. Study 1 showed that children were very positive about Black peers: Children liked them more and said they were smarter than White peers. These findings were somewhat unexpected, as children often express liking White people more (Raabe and Beelmann 2011; Sierksma et al. 2018) although previous work also showed that Dutch children sometimes evaluate Black peers relatively positively (de Bruijn et al. 2020). Our sample in Study 1 consisted mostly of 10–12‐year‐olds, and children this age often are less inclined to express explicit ethnic and racial bias because they are aware of anti‐discrimination norms (Apfelbaum et al. 2008). Moreover, we assessed attitudes and stereotypes with forced‐choice measures, which might have amplified this concern (i.e., children could easily deduct what we tried to measure). For Study 2, we therefore recruited a larger age range (7–12 years) and used Likert scales (i.e., asking children about one target at the same time) to assess ethnic attitudes and intelligence stereotypes. For Study 2, we preregistered to only include native Dutch children in our final analyses, as children with other ethnic backgrounds might help Black and White peers in different ways (note that we did not have sufficient power to test for these differences).

### 3.1 Method

#### 3.1.1 Participants

Based on summary‐statistics‐based power analysis for mixed‐effects modeling of nested data (Murayama et al. 2022), we decided to aim for 90 children per within‐subjects construct (i.e., ethnic group, difficulty of the puzzle), resulting in a minimum total sample size of 180 children. Data collection took place in the same science museum as in Study 1, and we included all children who wanted to participate during our time slot (*N* = 237). As preregistered, we excluded children who did not finish (*n* = 8) or whose parents indicated they were not native Dutch. Our final sample (*N* = 181) included 7–12‐year‐old children with a mean age of 9.26 (SD = 1.51; 7 years: 24, 8 years: 40, 9 years: 39, 10 years: 40, 11 years: 19, 12 years: 19) and 90 boys and 91 girls. The median annual gross household income fell in the category of €70.500–€94.000 (27.8%) and ranged from €11.500–€94.000 or more (12.7% did not disclose this information). The modal educational level of parents was higher professional education (Parent 1: 43.6%, Parent 2: 35%; about 2% did not report this information). This study was preregistered (see https://osf.io/g29hy).

#### 3.1.2 Measures^2^

##### 3.1.2.1 Helping and Help Preference

The helping task and items assessing children's own help preference were identical to Study 1.

##### 3.1.2.2 Ethnic Attitudes and Intelligence Stereotypes

To measure ethnic attitudes, we showed children six White children and six Black children and they indicated for each child how much they liked them (5‐point smiley face scale, with one indicating a frowning face and five indicating a smiling face; pictures from LoBue and Thrasher 2014). For intelligence stereotypes, we used the same procedure, but this time we asked how smart they thought the child was. For the final measures, we computed a difference score (ratings of White targets–ratings of Black targets; higher scores representing more favorable attitudes toward White peers).

### 3.2 Results

#### 3.2.1 Preregistered Analyses

##### 3.2.1.1 Ethnicity and Difficulty

A mixed linear model (see Table 1) showed a main effect of task difficulty (*p* < 0.001; easy puzzles *M* = 2.57, SD = 0.77; difficult puzzles *M* = 1.16, SD = 1.13). Just as in Study 1, children were less likely to give mastery‐oriented help for difficult puzzles compared to easy puzzles. Ethnicity of the recipient of help did not influence the type of help children gave (*p* = 0.139), and there was no interaction of ethnicity and puzzle difficulty (*p* = 0.181). Results were not influenced by children's age (all *p*s > 0.064; see Supporting Information for details).

There was a significant interaction for children's gender and difficulty (*β* = 0.10, SE = 0.04, *p* = 0.018, 95% CI [0.02, 0.19]. Simple effects analyses showed that both girls (*β* = 0.49, SE = 0.06, *p* < 0.001, 95% CI [0.36, 0.61]) and boys (*β* = 0.70, SE = 0.06, *p* < 0.001, 95% CI [0.57, 0.82]) provided less mastery‐oriented help when puzzles were difficult, but that this tendency was stronger for boys. There were no other significant effects for gender (all *p*s > 0.320; see Supporting Information for details).

#### 3.2.2 Exploratory Analyses

##### 3.2.2.1 Ethnic Attitudes and Stereotypes

Similar to Study 1, children showed little ethnic bias. They liked Black (M = 3.80, SD = 0.69) and White (*M* = 3.70, SD = 0.78) children equally, *t*(178) = −1.54, *p* = 0.125, *d* = −0.12, 95% CI [−0.26, 0.03]. In addition, they thought Black children (*M* = 3.63, SD = 0.63) were smarter than White children (*M* = 3.29, SD = 0.83), *t*(178) =−5.07, *p* < 0.001, *d* = 0.38, 95% CI [−0.53, −0.23].

There were no main (*β* = −0.04, SE = 0.03, *p* = 0.154, 95% CI [−0.10, 0.02]), or two‐way interactions for children's ethnic attitudes (with either task difficulty, *β* = −0.01, SE = 0.05, *p* = 0.861, 95% CI [−0.10, 0.08], or recipients’ ethnicity, *β* = −0.03, SE = 0.02, *p* = 0.180, 95% CI [−0.06, 0.01]), but the three‐way interaction with task difficulty, recipient's ethnicity, and ethnic attitudes was significant (*β* = 0.04, SE = 0.02, *p* = 0.038, 95% CI [0.002, 0.07]). Simple slope analyses for ethnic attitudes (1 SD below and above the mean) showed that the two‐way interaction between task difficulty and recipients ethnicity was significant for children who were positive about Black peers (i.e., scoring low on ethnic attitudes, *β* = −0.05, SE = 0.02, *p* = 0.025, 95% CI [−0.10, −0.01]), but not for children who were less positive about Black peers (i.e., scoring high on ethnic attitudes, *β* = 0.02, SE = 0.02, *p* = 0.487, 95% CI [−0.03, 0.06]). We then continued with simple effects analyses for children who were positive about Black peers (i.e., scoring low on ethnic attitudes). Results showed that when puzzles were difficult, children gave less mastery‐oriented help to Black recipients compared to White recipients, (*β =* 0.11, SE = 0.04, *p* = 0.003, 95% CI [0.04, 0.18]) but they did not differentiate when puzzles were easy (*β* = −0.00, SE = 0.04, *p* = 0.975, 95% CI [−0.07, 0.07]). Thus, in line with our hypothesis, children provided less mastery‐oriented help to Black recipients compared to White recipients when tasks were difficult, but only when they reported liking Black peers more than White peers.

Stereotypes about intelligence did not influence children's helping behavior (all *p*s > 0.132; see Supporting Information for details).

##### 3.2.2.2 Help Preferences

For easy puzzles, children again mostly wanted to receive mastery‐oriented help (94.4%, binomial test *p* < 0.001, 2 children did not answer this question). For difficult puzzles, there was no clear preference, 49.2% mastery‐oriented, 50.8% outcome‐oriented, 2 missing; binomial test, *p* = 0.881). Children preferences significantly influenced their helping behavior: Children who preferred mastery‐oriented help provided more mastery‐oriented help on difficult puzzles (*β* = 0.39, SE = 0.06, *p* < 0.001, 95% CI [0.27, 0.50]) and easy puzzles (*β* = 0.41, SE = 0.09, *p* < 0.001, 95% CI [0.24, 0.58]). Children's help preferences did not influence how they helped recipients of different ethnicities (i.e., no interactions, all *p*s > 0.437).

### 3.3 Discussion

Study 2 largely replicates the findings of Study 1: children were less inclined to provide mastery‐oriented help when tasks were difficult as opposed to easy. Moreover, when children were relatively positive about Black peers, they gave them less mastery‐oriented help on difficult tasks compared to White peers. Children were again very positive about Black peers compared to White peers, suggesting the age groups and forced‐choice nature of our measures in Study 1 were not the main reason for these preferences. Both Studies 1 and 2 yielded results that, in part, confirmed our hypothesis for ethnicity. But the effects were small and only emerged for a subset of children (who were very positive about Black peers). Because Studies 1 and 2 had the same design, we combined the data and performed a highly powered test of the impact of task difficulty and ethnicity across all children.

## 4 Internal Meta‐Analysis Study 1 and 2

We combined the data of Studies 1 and 2 including only Dutch children (*N* = 306 children). We could not analyze the influence of ethnic attitudes and stereotypes about intelligence because Studies 1 and 2 used different scales (i.e., forced choice versus Likert scales). The full model, including random slopes for difficulty and ethnicity and a random intercept, did not converge. We therefore compared the fit of a model including only a random slope for ethnicity and only a random slope for difficulty, and the fit was better for the latter and thus we proceeded with that model. There was a main effect of task difficulty: Children provided less mastery‐oriented help when tasks were difficult compared to easy (*β* = 0.57, SE = 0.04, *p* < 0.001, 95% CI [0.50, 0.64]). There was no main effect of ethnicity (*β* = 0.02, SE = 0.01, *p* = 0.137, 95% CI [−0.01, 0.05]), and the interaction between recipient's ethnicity and task difficulty was marginally significant (*β* = −0.02, SE = 0.01, *p* = 0.090, 95% CI [−0.05, 0.00]).

**FIGURE 2:**
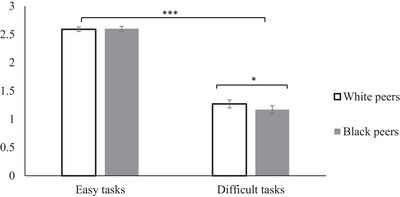
Number of times children provided mastery‐oriented help (vs. outcome‐oriented help), internal meta‐analysis Studies 1 and 2. Note: Error bars represent standard error. ***p<0.001, *p<0.05.

Follow‐up analyses showed that when tasks were difficult, children provided less mastery‐oriented help to Black compared to White peers (*β* = 0.04, SE = 0.02, *p* = 0.025, 95% CI [0.01, 0.08]). When tasks were easy, children helped White and Black recipients similarly (*β* = −0.00, SE = 0.02, *p* = 0.885, 95% CI [−0.04, 0.03]; see Figure 2).

There was a main effect of age (*β* = 0.08, SE = 0.03, *p* = 0.009, 95% CI [0.02, 0.14]) but no interactions (all *p*s > 0.092), and gender did not influence the results (all *p*s > 0.135; see Supporting Information for details).

The internal meta‐analysis thus shows that children are less inclined to provide mastery‐oriented help on difficult tasks and that this tendency was more pronounced when recipients were Black. Studies 1 and 2 further showed that children were less likely to provide mastery‐oriented help to Black peers when they liked this group. Overall, these findings suggest that liking an ethnic group (but not stereotypes about intelligence) can cause children as young as 7 years to help in less empowering ways, similar to adults (Cuddy et al. 2007). To better understand if indeed these positive feelings toward ethnic groups’ children do not drive differential helping, we conducted a third study in which we examined the type of help children provided to a different ethnic group: Middle‐Eastern recipients. This is a group that Dutch adults and children often view negatively (de Bruijn et al. 2020; Hagendoorn 2001).

## 5 Study 3

### 5.1 Method

#### 5.1.1 Participants

Based on a priori power analysis, we aimed to include a minimum of 180 children. We started data collection at the science museum (*n* = 171), but because we did not reach our final sample size, we also recruited schools, and some children were tested there (*n* = 25). A total of 196 7–12‐year‐old children participated (one child was excluded because they did not finish). As preregistered, we included only Dutch children in our final analyses. The final sample of 182 children had a mean age of 9.39 (SD = 1.61; 7 years: 25, 8 years: 37, 9 years: 33, 10 years: 32, 11 years: 28, 12 years: 23, 5 parents did not report age) and 56.6% was a boy and 43.4% a girl. The median annual gross household income fell in the category €70.500–€94.000 (16.4%) and ranged from €11.500 to €94.000 or more (19.2% did not report income). The modal educational level of parents was a university degree (Parent 1 39%, Parent 2: 32.4%; about 4% did not report this information). This study was preregistered (see https://osf.io/sh8ua).

#### 5.1.2 Design and Procedure

Design of the study was similar to Studies 1 and 2. To manipulate the ethnicity of the recipient of help we included pretested pictures of Middle‐Eastern and White children (de Bruijn et al. 2020). We selected four children per racial group and gender (16 trials total; 8 per child). Children thus helped a total of eight targets, which was less than in Studies 1 and 2 because we did not have a sufficient number of pretested pictures that were of good quality. All other measures were identical to Study 2.

#### 5.1.3 Analyses

We preregistered to use sum scores as our main dependent variable for helping. However, some counterbalancing errors occurred (see Supporting Information for details), and therefore not all children helped the exact same number of trials in each condition (i.e., sum scores could not be used because there were missing scores on some trials). We therefore analyzed children's helping as a mean score.

### 5.2 Results

#### 5.2.1 Preregistered Analyses

##### 5.2.1.1 Difficulty and Ethnicity

A linear mixed effect model (see Table 1) showed a main effect for task difficulty: Children provided less mastery‐oriented help when puzzles were difficult compared to when puzzles were easy (*p* < 0.001; easy puzzles, *M* = 0.87, SD = 0.31; difficult puzzles, *M* = 0.39, SD = 0.42). There was no main effect for recipient ethnicity (*p* = 0.441) and the interaction for ethnicity and task difficulty was also not significant (*p* = 0.630).

There was a main effect of age (*β* = 0.16, SE = 0.03, *p* < 0.001, 95% CI [0.10, 0.22]), suggesting older children provided more mastery‐oriented help, but no interaction effects (*p*s > 0.199). Children's gender did not impact how they helped (*p*s > 0.216; see Supporting Information for details).

#### 5.2.2 Exploratory Analyses

##### 5.2.2.1 Ethnic Attitudes and Intelligence Stereotypes

On average, children liked White children (*M* = 3.96, SD = 0.68) more than Middle‐Eastern children (*M* = 3.55, SD = 0.77), *t*(179) = 8.03, *p* < 0.001, *d* = 0.60, 95% CI [0.44, 0.76]). They also thought White children (*M* = 3.65, SD = 0.69) were smarter than Middle‐Eastern children (*M* = 3.43, SD = 0.74), *t*(179) = 3.83, *p* < 0.001, *d* = 0.29, 95% CI [0.14, 0.43]). There was a main effect for ethnic attitudes (*β* = −0.08, SE = 0.03, *p* = 0.026, 95% CI [−0.15, −0.01]), suggesting that liking White peers (vs. Middle‐Eastern peers) more was related to providing less mastery‐oriented help in general. However, interactions were not significant (all *p*s > 0.088). Stereotypes about intelligence did not influence children's helping (all *p*s > 0.242; see Supporting Information for details).

##### 5.2.2.2 Children's Own Help Preferences

Again, children preferred to receive mastery‐oriented help for easy puzzles (90.7%; binomial test, *p* < 0.001), but they were split when it concerned difficult puzzles: 50.5% preferred mastery‐oriented help, 49.5% wanted outcome‐oriented help (binomial test, *p* = 0.886). Children who preferred mastery‐oriented help provided more mastery‐oriented help on both easy puzzles (*β* = 0.54, SE = 0.07, *p* < 0.001, 95% CI [0.40, 0.68]) and difficult puzzles (*β* = 0.36, SE = 0.06, *p* < 0.001, 95% CI [0.25, 0.47]). Children's preferences did not influence how they helped peers of different ethnicities (i.e., no interactions, all *p*s > 0.595).

Taken together, Study 3 replicates the finding that children provide less mastery‐oriented help on difficult tasks. Whether recipients of help were White or Middle‐Eastern did not impact the type of help they provided, suggesting that the findings of Studies 1 and 2 were particular for the ethnic group children helped.

## 6 General Discussion

Educators and parents often assume help exchanges benefit children's development and learning (Tenenbaum et al. 2020). Here, we show that children do not always provide the type of help that is beneficial for learning: Children provided less mastery‐oriented help when tasks are difficult and when recipients of help belonged to ethnic groups they liked. These results have important implications for promoting exchanges of beneficial help early in life.

### 6.1 Theoretical Implications: Task Difficulty

Children often ask peers for help in the classroom, especially when they struggle with tasks (Altermatt et al. 2002; Ryan and Shim 2012). Our work shows that peers might then not always provide the most beneficial help: In all three studies, children gave recipients less mastery‐oriented help compared to outcome‐oriented help when tasks were difficult. Earlier studies already showed that children take into account how hard things are for others when they help (i.e., whether to help or how much help to provide; Baer and Friedman 2018; Bennett‐Pierre et al. 2018; Bridgers et al. 2020; Gelman et al. 2013; Ronfard and Harris 2018; Sierksma 2023a; Sierksma and Shutts 2021). And children provide less mastery‐oriented help when they perceive recipients as incompetent (Sierksma 2023a). Thus, uncertainty about whether recipients will profit from being challenged, whether based on person attributes (i.e., Sierksma 2023a) or features that are inherent to the task (i.e., the current work), reduces the provision of mastery‐oriented help in children.

Often, giving more help to those that work on harder tasks is beneficial, because it makes sure that recipients who need more help also get it. Yet, when the type of help provided does not benefit recipients in the long term, it might back‐fire. First, if children systematically refrain from providing mastery‐oriented help on harder tasks this can directly impact learning and achievement at school (Ryan and Shim 2012; Schenke et al. 2015). That is, children do not acquire new skills from just getting the correct answer or someone doing the task for them. Second, providing more outcome‐oriented help can lead to dependency of recipients in the domain of help (Nadler 2002). After all, when children do not acquire new skills to understand and solve challenging tasks, they might require and seek help over and over again. Third, differential helping can have negative downstream consequences for recipient's self‐views and motivation. Receiving help can threaten feelings of autonomy and control (Ryan et al. 2001; Ryan and Deci 2018) and reduce children's motivation to work on challenging tasks (Leonard 2021; Sierksma and Brummelman 2025). Outcome‐oriented help could signal to children that others have little confidence in their abilities, as it suggests that helpers assume long‐term mastery is unlikely. Indeed, children think people who are less smart are more likely to get outcome‐oriented help (Sierksma 2023a). As such, children's differential helping could negatively impact academic self‐views and in turn lower motivation for school tasks (Wu et al. 2021).

What can we do to stimulate children to provide more mastery‐oriented help when things get hard? Answering this question requires that we first understand what drives children to help this way. One likely mechanism is that children are motivated to reduce people's needs in the short run rather than in the long term. Teaching children the value of mastery‐oriented help could change that: Teaching practices that emphasize learning and growth tend to promote mastery learning goals (Meece et al. 2006) and seeking mastery‐oriented help (Schenke et al. 2015). In all three studies, children helping aligned with their own help preferences. Mastery‐oriented teaching practices might thus also stimulate children to help others achieve mastery.

### 6.2 Theoretical Implications: Interethnic Helping

In‐group bias and prejudice are often assumed to thwart people's inclination to help outgroups (Moran et al. 2024; Sierksma 2023b). And adults sometimes provide less mastery‐oriented help to lower status outgroups (Nadler 2002). Our results, in part, provide support for such tendencies in children: The internal meta‐analysis of Studies 1 and 2 showed that when tasks were difficult, Dutch children gave Black peers less mastery‐oriented help than White peers. However, it was not dislike or negative stereotypes that seemed to motivate children to help this way. Rather, children liked Black peers more (Study 1) or just as much (Study 2) as White peers, and this positive attitude was related to providing less mastery‐oriented help to Black versus White peers. Conversely, when children did not like an ethnic group (Study 3, Middle‐Eastern), they also did not provide less mastery‐oriented help to them.

Liking other ethnic groups thus seems to promote the provision of less beneficial types of help in children. Why? The Stereotype Content Model (Cuddy et al. 2007; Fiske et al. 2002) postulates that warmth and competence are central to person perception and, in particular, perceptions of warmth can trigger feelings of pity and paternalistic helping (i.e., restricting a person's development or choice because the helper believes this is in the recipients’ best interest). Perhaps our ethnic attitude measure picked up such feelings of warmth, and children might thus have provided less mastery‐oriented help to Black peers because they felt pity for them. Other mechanisms could also explain the findings, however. Social desirability, for example, could underlie some of the findings, such that children thought it was more socially desirable to express positive attitudes about Black peers and to give outcome‐oriented help. To better understand why liking impacted inter‐ethnic helping, an important step forward is to examine how Dutch children perceive different ethnic groups and ask them to explain why they helped this way.

Across all three studies, children's endorsement of stereotypes about intelligence was not related to their helping. This is not in line with previous work in which Dutch children (mean age of 11 years) helped Black peers more when they endorsed a stereotype that this group was less intelligent (Sierksma et al. 2018) and also seems contrary to our idea that uncertainty about whether mastery‐oriented help is useful for recipients, reduces it. How can we explain that stereotypes about intelligence did not impact children's helping? First, we measured children's endorsement of societal stereotypes by letting them evaluate unfamiliar exemplars of an ethnic category, and we did not ask them how intelligent recipients of help were. Children can be familiar with stereotypes, but that does not always mean they apply them to exemplars of a group (Degner and Wentura 2010; Legaspi et al. 2023). Second, perhaps children's age can explain this apparent discrepancy: Children often express biased attitudes at a young age (Raabe and Beelmann 2011; Shutts 2015), but their knowledge of stereotypes might emerge later because stereotypes are more complex and multifaceted (Mackie et al. 1996; Sierksma 2023b). Indeed, in the current study, we found little evidence of application of stereotypes about intelligence for Black peers (but children did endorse stereotypes about intelligence for Middle‐Eastern peers, a group for which negative stereotypes are probably much more salient in Dutch society; see de Bruijn et al. 2020). We thus speculate that there might be a developmental shift in what drives differential helping in children: Young children's ethnic helping might be guided by concern for others, and positive ethnic attitudes amplify this concern, whereas older children help based on their knowledge of stereotypes (Sierksma et al. 2018). Future studies could examine these explanations by including a larger age range, using longitudinal methods, and by assessing how intelligent children think each recipient of help is.

Taken together, these findings provide new insight into why children do not always help equally in interethnic contexts. It should be noted that the findings were not entirely consistent across Studies 1 and 2, and effects were small and thus future efforts are needed to replicate and extend the findings. If replicated, we can conclude that when children like ethnic groups, or sympathize with them, this leads them to provide help that hampers the development of new skills which, in the long term, can perpetuate competence‐based inequality.

How can we stimulate children to provide more mastery‐oriented help to *all* peers? If our liking measure is related to feelings of pity or sympathy, then we might have to invest more in how children perceive groups in their society. Marginalized or lower status groups are often portrayed as weak and vulnerable (Bauer et al. 2021; Brannon 2023; Silverman et al. 2023). New perspectives in the social sciences challenge this view and argue that people belonging to marginalized groups also endured challenges, built resilience, and acquired a specialized skill set along the way (Ellis et al. 2022). Moving away from a deficit perspective toward a strength‐based approach is important in itself (Brummelman et al. 2024) and could also promote children to provide mastery‐oriented help to peers who belong to lower status groups in their society.

### 6.3 Strengths, Limitations, and Future Directions

Strengths of our research include internal replications, an experimental design that allows establishing causality, and inclusion of a large age range. Our research also has limitations.

Several factors in our design warrant further scrutiny. First, we recommend that future studies counterbalance the colors linked to the types of help (i.e., we only included orange and green). Second, children could provide two particular types of help (hints, correct answers) on a restricted set of tasks (easy, difficult world puzzles). Next steps include looking at how children provide different types of help (i.e., asking questions and taking over) in other academic domains (e.g., math) and across tasks that vary in difficulty. At what point would children switch from mastery to outcome‐oriented help? And do children provide more outcome‐oriented help for academic domains that are often perceived as harder (e.g., math)? Third, an important next step is to conduct field studies. Observing how peer‐to‐peer‐helping naturally unfolds in classrooms can shed new light on the role that task‐ and recipient‐related factors play in *how* children at different ages help spontaneously (e.g., do children help their friends differently than disliked peers?).

Our findings support the idea that positive attitudes underlie providing less mastery‐oriented help to ethnic groups, but we did not directly assess this. It is therefore important that future work assesses how children feel about the groups and ask them to explain why they provide certain types of help. Such work should also aim to include a more diverse sample of children. Our studies included predominantly White Dutch children from relatively high social economic backgrounds. Children who belong to non‐White or lower social economic backgrounds might not feel sorry for lower status groups or might even be more motivated to help them advance.

## 7 Conclusion

Children are motivated to help others and often their help benefits recipients. Yet, sometimes help has negative unintended consequences. Here, we show that these unintended consequences can emerge when tasks are difficult and recipients belong to like ethnic groups. A deeper understanding of how and why children distribute different types of help can aid us in promoting exchanges of help that foster learning for all peers.

## Author Contributions

Both authors were involved in conceptualization and designing the methodology. J.S. was additionally responsible for data curation, formal analysis, funding acquisition, project administration, and writing (original draft and review and editing). A.P. was also involved in writing (review and editing).

## Ethics Statement

All three studies were approved by the ethical review board of Utrecht University (Study 1: 22‐0279; Study 2: 23‐0146; Study 3: 23‐0290).

## Conflicts of Interest

The authors declare no conflict of interest.

## Supporting information




**Supporting File 1**: desc70071‐Sup‐0001‐SuppMat.docx

## Data Availability

The data that support the findings of this study are openly available in open science framework at https://osf.io/pmzct/?view_only=d494590646cf41299aa727522309e70a.

## Endnotes

1 Note that we preregistered to use logistic linear models, but because we used sum scores for the type of help the data no longer have a binomial structure.

2 Studies 2 and 3 included additional measures related to children's learning goals. We preregistered exploratory analyses for these measures. We decided to not include the measures here but use them for another project.
