# Supplementary material for: Mastery‐Oriented or Outcome‐Oriented Help? How Recipient Ethnicity and Task Difficulty Shape Children's Helping Behavior
Source: Dev Sci. 2025 Sep 8;28(6):e70071. doi: 10.1111/desc.70071 (PMC12417624; doi:10.1111/desc.70071)
Supplement: Supplementary file 1 — Supporting File 1: desc70071‐Sup‐0001‐SuppMat.docx [file DESC-28-e70071-s001.docx]

**Supplemental materials**

**Full statistics for exploratory analyses in all three studies**

**Study 1**

**Ethnicity and difficulty.** Children’s age did not influence helping (main effect: *β*=0.07, SE=0.04, *p*=.053, 95% CI [-0.00, 0.01] ; interaction with difficulty, *β*=-0.02, SE=0.05, *p*=.702, 95% CI [-0.12, 0.08]; interaction with ethnicity: *β*=0.01, SE=0.02, *p*=.495, 95% CI [-0.02, 0.04]; three-way interaction: *β*=-0.02, SE=0.02, *p*=.270, 95% CI [-0.05, 0.01]) and gender also did not impact how they helped (main effect: *β*=0.04, SE=0.03, *p*=.240, 95% CI [-0.03, 0.11]; interaction with difficulty, *β*=-0.04, SE=0.05, *p*=.411, 95% CI [-0.14, 0.06]; interaction with ethnicity: *β*=0.00, SE=0.02, *p*=.864, 95% CI [-0.03, 0.03]; three-way interaction: *β*=0.01, SE=0.02, *p*=.415, 95% CI [-0.02, 0.04].

**Stereotypes about intelligence.** Stereotypes about intelligence did not influence children’s helping behavior (main effect: *β*=-0.05, SE=0.04, *p*=.150, 95% CI [-0.12, 0.02]; interaction with difficulty, *β*=0.05, SE=0.05, *p*=.323, 95% CI [-0.05, 0.15]; interaction with ethnicity: *β*=-0.02, SE=0.02, *p*=.177, 95% CI [-0.05, 0.01]; three-way interaction: *β*=0.01, SE=0.02, *p*=.340, 95% CI [-0.01, 0.04]).

**Help preferences.** Children’s help preferences did not influence how they helped recipients of different ethnicities (easy puzzles: interaction with ethnicity, *β*=-0.01, SE=0.03, *p*=.777, 95% CI [-0.06, 0.04], interaction with difficulty and ethnicity, *β*=0.00, SE=0.03, *p*=.937, 95% CI [-0.05, 0.05]; difficult puzzles, interaction with ethnicity, *β*=0.00, SE=0.02, *p*=.908, 95% CI [-0.03, 0.03], interaction with difficulty and ethnicity, *β*=-0.02, SE=0.02, *p*=.178, 95% CI [-0.05, 0.01] ).

**Study 2**

**Ethnicity and difficulty.** Results were not influenced by children’s age (main effect: *β*=0.06, SE=0.03, *p*=.064, 95% CI [-0.00, 0.12] ; interaction with difficulty, *β*=0.04, SE=0.04, *p*=.314, 95% CI [-0.04, 0.13]; interaction with ethnicity: *β*=0.02, SE=0.02, *p*=.331, 95% CI [-0.02, 0.05]; three-way interaction: *β*=0.03, SE=0.02, *p*=.121, 95% CI [-0.06, 0.01]).

There were no other significant effects for gender (main effect: *β*=0.00, SE=0.03, *p*=.898, 95% CI [-0.06, 0.07] ; interaction with ethnicity: *β*=0.02, SE=0.02, *p*=.320, 95% CI [-0.02, 0.06]; three-way interaction: *β*=-0.01, SE=0.02, *p*=.588, 95% CI [-0.04, 0.02]).

**Stereotypes about intelligence.** Stereotypes about intelligence did not influence children’s helping behavior (main effect: *β*=-0.00, SE=0.03, *p*=.996, 95% CI [-0.06, 0.06]; interaction with difficulty, *β*=-0.03, SE=0.05, *p*=.474, 95% CI [-0.12, 0.06]; interaction with ethnicity: *β*=0.03, SE=0.02, *p*=.132, 95% CI [-0.01, 0.06]; three-way interaction: *β*=0.02, SE=0.02, *p*=.280, 95% CI [-0.05, 0.02]).

**Help preferences.** Children’s help preferences did not influence how they helped recipients of different ethnicities (easy puzzles: interaction with ethnicity, *β*=-0.02, SE=0.04, *p*=.651, 95% CI [-0.09, 0.06], interaction with difficulty and ethnicity, *β*=0.00, SE=0.04, *p*=.975, 95% CI [-0.07, 0.07]; difficult puzzles, interaction with ethnicity, *β*=0.01, SE=0.02, *p*=.437, 95% CI [-0.02, 0.05], interaction with difficulty and ethnicity, *β*=0.01, SE=0.02, *p*=.600, 95% CI [-0.02, 0.04] ).

**Internal meta-analysis**

**Ethnicity and difficulty.** There was a main effect of age (*β*=0.08, SE=0.03, *p*=.009, 95% CI [0.02, 0.14]) but no interactions (interaction with difficulty, *β*=-0.01, SE=0.04, *p*=.685, 95% CI [-0.06, 0.08]; interaction with ethnicity: *β*=0.01, SE=0.01, *p*=.593, 95% CI [-0.02, 0.03]; three-way interaction: *β*=-0.02, SE=0.01, *p*=.092, 95% CI [-0.05, 0.00]). Gender did not influence the results (main effect: *β*=-0.02, SE=0.02, *p*=.389, 95% CI [-0.03, 0.07]; interaction with difficulty, *β*=-0.05, SE=0.04, *p*=.135, 95% CI [-0.02, 0.12]; interaction with ethnicity: *β*=0.01, SE=0.00, *p*=.319, 95% CI [-0.01, 0.04]; three-way interaction: *β*=0.00, SE=0.01, *p*=.970, 95% CI [-0.03, 0.03]).

**Study 3**

**Ethnicity and difficulty.** There was a main effect of age (*β*=0.16, SE=0.03, *p*<.001, 95% CI [0.10, 0.22]) but no interaction effects (interaction with difficulty, *β*=-0.03, SE=0.05, *p*=.473, 95% CI [-0.12, 0.06]; interaction with ethnicity: *β*=-0.01, SE=0.02, *p*=.735, 95% CI [-0.05, 0.03]; three-way interaction: *β*=-0.02, SE=0.02, *p*=.199, 95% CI [-0.06, 0.01]). Children’s gender did not impact how they helped (main effect: *β*=-0.04, SE=0.03, *p*=.216, 95% CI [-0.10, 0.02]; interaction with difficulty, *β*=0.02, SE=0.05, *p*=.700, 95% CI [-0.07, 0.11]; interaction with ethnicity: *β*=-0.00, SE=0.02, *p*=.981, 95% CI [-0.04, 0.04]; three-way interaction: *β*=-0.00, SE=0.02, *p*=.892, 95% CI [-0.04, 0.03].

**Stereotypes about intelligence**. There was a main effect for ethnic attitudes (*β*=-0.08, SE=0.03, *p*=.026, 95% CI [-0.15, -0.01]) but interactions were not significant (interaction with difficulty, *β*=0.06, SE=0.05, *p*=.228, 95% CI [-0.04, 0.15]; interaction with ethnicity: *β*=-0.04, SE=0.02, *p*=.088, 95% CI [-0.08, 0.01]; three-way interaction: *β*=-0.03, SE=0.02, *p*=.193, 95% CI [-0.01, 0.06]. Stereotypes about intelligence did not influence children’s helping (main effect: *β*=-0.02, SE=0.03, *p*=.584, 95% CI [-0.08, 0.05]; interaction with difficulty, *β*=0.03, SE=0.05, *p*=.580, 95% CI [-0.07, 0.12]; interaction with ethnicity: *β*=-0.02, SE=0.02, *p*=.242, 95% CI [-0.07, 0.02]; three-way interaction: *β*=-0.01, SE=0.02, *p*=.592, 95% CI [-0.03, 0.05].

**Help preferences.** Children’s preferences did not influence how they helped peers of different ethnicities (easy puzzles: interaction with ethnicity, *β*=-0.02, SE=0.04, *p*=.676, 95% CI [-0.09, 0.06], interaction with difficulty and ethnicity, *β*=0.01, SE=0.03, *p*=.815, 95% CI [-0.06, 0.07]; difficult puzzles, interaction with ethnicity, *β*=0.01, SE=0.02, *p*=.703, 95% CI [-0.03, 0.05], interaction with difficulty and ethnicity, *β*=-0.01, SE=0.02, *p*=.595, 95% CI [-0.05, 0.03]).

**Results for whether children’s helping differed from chance**

| *Study* | Black targets,  hard puzzle | Black targets,  easy puzzle | White targets,  hard puzzles | White targets,  easy puzzles |
| --- | --- | --- | --- | --- |
| Study 1 | t=-3.10, *p*=.002 | t=18.73, *p<*.001 | t=-2.20, *p*=.03 | t=18.08, *p*<.001 |
| Study 2 | t=-4.79, *p*<.001 | t=19.26, *p*<.001 | t=--3.28, *p*<.001 | t=18.42, *p*<.001 |
| Study 3 | t=-3.39, *p*<.001 | t=16.81, *p*<.001 | t=-3.52, *p*<.001 | t=15.14, *p*<.001 |
| *Note*. Results for t-tests against chance, Study 1 and 2 chance was 1.5, for Study 3 chance was 0.5 | | | | |

**Study 3**

**Counterbalancing errors.** In Study 3 children helped on a total of 8 trials. And we randomly showed children 8 different targets: 2 were White and worked on an puzzle, 2 were Black and worked on an easy puzzle, 2 were White and worked on a difficult puzzle, and 2were Black peers and worked on a difficult puzzle. We also made sure that the photos of targets were sometimes matched to a difficult puzzle and sometimes matched to a hard puzzle. So some children saw a White child work was working on an easy puzzle and other participants saw the same White target who worked on a difficult puzzle. For boys counterbalancing was correct. For girls, there was a programming mistake. 50% of the girls saw the correctly counterbalanced set, but 25% of girls helped 6 targets with easy puzzles (3 White and 3 black) and 2 with difficult puzzles (1 White, 1 Black), and the other 25% helped 6 targets with difficult puzzles (3 White and 3 black) and 2 with easy puzzles (1 White, 1 Black). Although this error resulted in an imbalance in terms of task difficulty, children never saw the same target twice. As such, this error did not affect the results but prevented us from using sum scores.
